# Pre-plaque Aß-Mediated Impairment of Synaptic Depotentiation in a Transgenic Rat Model of Alzheimer’s Disease Amyloidosis

**DOI:** 10.3389/fnins.2019.00861

**Published:** 2019-08-14

**Authors:** Yingjie Qi, Igor Klyubin, Neng-Wei Hu, Tomas Ondrejcak, Michael J. Rowan

**Affiliations:** ^1^Department of Pharmacology & Therapeutics, Institute of Neuroscience, Trinity College Dublin, Dublin, Ireland; ^2^Department of Physiology and Neurobiology, Zhengzhou University School of Medicine, Zhengzhou, China

**Keywords:** soluble amyloid beta, synaptic plasticity, hippocampus, novelty exploration, depotentiation, apical dendrites, basal dendrites, Alzheimer’s disease

## Abstract

How endogenously produced soluble amyloid ß-protein (Aß) affects synaptic plasticity in vulnerable circuits should provide insight into early Alzheimer’s disease pathophysiology. McGill-R-Thy1-APP transgenic rats, modeling Alzheimer’s disease amyloidosis, exhibit an age-dependent soluble Aß-mediated impairment of the induction of long-term potentiation (LTP) by 200 Hz conditioning stimulation at apical CA3-to-CA1 synapses. Here, we investigated if synaptic weakening at these synapses in the form of activity-dependent persistent reversal (depotentiation) of LTP is also altered in pre-plaque rats *in vivo*. In freely behaving transgenic rats strong, 400 Hz, conditioning stimulation induced stable LTP that was NMDA receptor- and voltage-gated Ca^2+^ channel-dependent. Surprisingly, the ability of novelty exploration to induce depotentiation of 400 Hz-induced LTP was impaired in an Aß-dependent manner in the freely behaving transgenic rats. Moreover, at apical synapses, low frequency conditioning stimulation (1 Hz) did not trigger depotentiation in anaesthetized transgenic rats, with an age-dependence similar to the LTP deficit. In contrast, at basal synapses neither LTP, induced by 100 or 200 Hz, nor novelty exploration-induced depotentiation was impaired in the freely behaving transgenic rats. These findings indicate that activity-dependent weakening, as well as strengthening, is impaired in a synapse- and age-dependent manner in this model of early Alzheimer’s disease amyloidosis.

## Introduction

There is great interest in understanding how different forms of synaptic plasticity contribute to normal brain function and how disruption of these physiological processes may underlie key aspects of the pathophysiology of neurodegenerative diseases. Although much research has elucidated the physiological significance of long-term potentiation (LTP) in memory mechanisms and have implicated impairments of LTP in cognitive impairment, in particular in Alzheimer’s disease (AD) models [for review see Spires-Jones and Knafo (2012), Puzzo et al. (2015)], much less is known regarding activity-dependent persistent LTP reversal (depotentiation).

Usually, LTP decay/reversal is an active process and mechanisms underlying depotentiation are believed to play important roles in the time- and state- dependent erasure of certain forms of memory. Behaviourally induced depotentiation at CA3-to-CA1 hippocampal synapses occurs within a specific time-window when an animal acquires new information (Xu et al., 1998; Manahan-Vaughan and Braunewell, 1999; Abraham et al., 2002; Straube et al., 2003; Collingridge et al., 2010; Qi et al., 2013). Similar depotentiation can be induced with low frequency electrical conditioning stimulation both *in vivo* and *in vitro* (Staubli and Lynch, 1990; Fujii et al., 1991; Bashir and Collingridge, 1994; Migues et al., 2016).

Exogenous application of certain soluble aggregates of the AD protein amyloid ß (Aß), in particular certain soluble aggregates/oligomers of Aß (Aßo), potently inhibit LTP in wild-type (WT) rodents (Cullen et al., 1997; Lambert et al., 1998; Hu et al., 2008; Klyubin et al., 2014). Recently, we reported that prior to the deposition of fibrillar Aß in plaques in APP transgenic (TG) rats, endogenously produced Aß causes an age-dependent disruption of LTP induced by 200 Hz at CA1 apical synapses, the deficit being transiently rescued by subacute administration of agents that lower soluble Aß (Qi et al., 2014). An accumulation of Aß oligomers accompanies the impairment of NMDA receptor-dependent LTP (Qi et al., 2014; Zhang D. et al., 2017) and selective reduction in NMDA, but not AMPA, receptor-mediated baseline synaptic transmission (Qi et al., 2014). The LTP deficit in pre-plaque transgenic rats appears to be mediated by an age-dependent pro-inflammatory milieu in the hippocampus (Leon et al., 2010; Hanzel et al., 2014; Iulita et al., 2014; Qi et al., 2018) driven by Aß oligomer binding to cellular prion protein and glutamate acting at metabotropic glutamate receptor 5 (Zhang D. et al., 2017). Somewhat similarly, an early intracellular buildup of Aß oligomers correlates with impairment of hippocampus-dependent memory (Leon et al., 2010; Hanzel et al., 2014; Iulita et al., 2014) in the absence of observable synaptic structural change (Martino Adami et al., 2017).

Whether or not other forms of synaptic plasticity such as depotentiation are also affected in an age-dependent manner is unknown. Based on our previous finding that exogenous application on an Aß-containing APP fragment induced depotentiation in a narrow time window (Kim et al., 2001) and Aß facilitates low frequency stimulation-induced long-term depression (LTD) in WT rats (Li et al., 2009; Hu et al., 2014), we predicted that the induction of depotentiation would be facilitated in TG rats. Furthermore, because the acute disruption of synaptic plasticity by exogenously applied Aßo is synapse-selective, with preferential vulnerability of apical over basal synapses to LTP inhibition (Hu et al., 2009), we expected that the disruption of LTP and depotentiation would be selective to apical, as opposed to basal, synapses in TG rats.

Consistent with a key role for endogenous Aßo in mediating LTP inhibition at apical synapses, an antibody that preferentially binds soluble aggregates of Aß over monomer reversed the deficit in freely behaving TG animals. Using a strong 400 Hz conditioning protocol we induced robust LTP, thereby allowing us to study depotentiation in these rats. To our surprise, we found that depotentiation was impaired both in freely behaving and anaesthetized TG animals. Interestingly, endogenous Aß-mediated inhibition of both LTP and depotentiation was restricted to apical synapses, with neither LTP nor depotentiation in TG rats being significantly altered at basal synapses. These findings indicate that in addition to synapse-selective deficits in LTP induction, the synaptic plasticity mechanisms for time-dependent weakening of previously strengthened synapses are also disrupted by Aßo in early pre-plaque AD amyloidosis.

## Materials and Methods

### Animals

Male TG rats (2.5–6 months old) expressing human APP751 with Swedish and Indiana mutations under the control of the murine Thy1.2 promoter (McGill-R-Thy1-APP) (Leon et al., 2010) and their age-matched WT littermates were genotyped commercially by Transnetyx (Cordova, TN, United States) using real time PCR. All experiments were carried out in accordance with the approval of the Health Products Regulatory Authority, Ireland, using methods similar to those described previously (Qi et al., 2014). Animals had free access to food and water and a 12-h lights on/off cycle.

### *In vivo* Surgery and Electrophysiology

For non-recovery experiments the rats were anaesthetized with urethane (1.5 g/kg, i.p.) and core body temperature was maintained at 37.5 ± 0.5°C. For recovery experiments the implantation procedure was comparable but carried out under anaesthesia using a mixture of ketamine and xylazine (80 and 8 mg/kg, respectively, i.p.) according to methods similar to those described previously (Qi et al., 2013). For the recovery experiments the rats were allowed at least 14 days after surgery before recordings began. These rats were housed individually in their home cages post-surgery between recording sessions.

**FIGURE 1:**
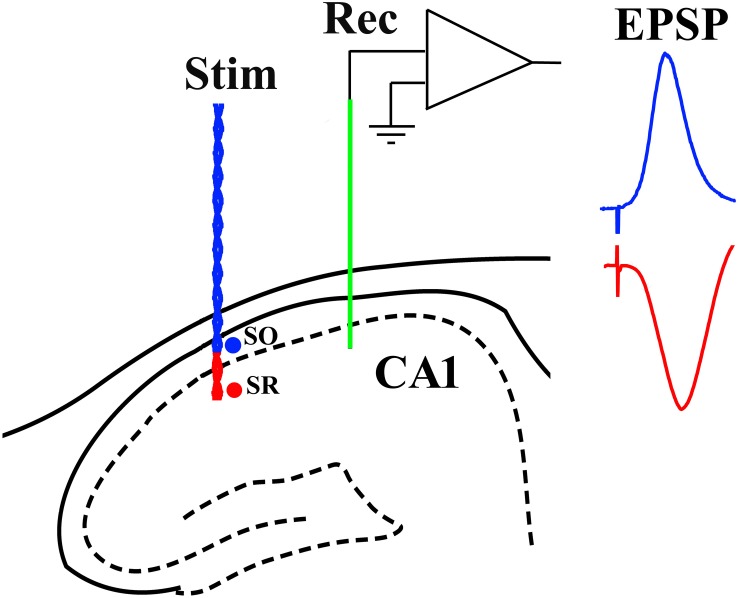
Schema of electrode configuration in the CA1 area of the dorsal hippocampus of the rat. A negative-going field EPSP (red trace), generated at apical synapses, was evoked by placing both a single-strand wire recording electrode (Rec, green) and a twisted-pair wire stimulation electrode (Stim, red) in stratum radiatum (SR). In contrast, a positive-going far-field EPSP (blue trace), generated at basal synapses, was evoked when the stimulation electrode was located in stratum oriens (SO) while the recording electrode (Rec, green) was implanted in SR.

Recording electrodes (Teflon-coated tungsten wire; external diameter 75 μm bipolar or 112 μm monopolar) were positioned in the stratum radiatum of area CA1. Similar wire electrodes were placed either in the stratum radiatum or stratum oriens to selectively stimulate either apical or basal synapses, respectively. Screw electrodes located over the contralateral cortex were used as reference and earth. The stimulation and recording electrodes were optimally located using a combination of physiological and stereotactic indicators. Field excitatory post-synaptic potentials (EPSPs) were recorded in the stratum radiatum of the dorsal hippocampus in response to stimulation of the ipsilateral stratum radiatum (apical synapses) or stratum oriens (basal synapses) (Figure 1). The recording site was located 3.8 mm posterior to bregma and 2.5 mm lateral to midline, and the stimulating site was located 4.6 mm posterior to bregma and 3.8 mm lateral to midline. The final depths of the electrodes were adjusted to optimize the electrically evoked EPSP and confirmed by post-mortem analysis. With the stimulation electrode in stratum oriens, the far-field EPSP from basal synapses was reversed in polarity because the recording electrode was located in the stratum radiatum (Leung et al., 2003).

Where necessary a stainless steel guide cannula (22 gauge, 0.7-mm outer diameter, length 13 mm) was implanted above the right lateral ventricle before the electrodes were implanted ipsilaterally. Injections were made via a Hamilton syringe which was connected to the internal cannula (28 gauge, 0.36 mm outer diameter). The injector was removed 1 min post-injection and a stainless steel plug was inserted. The position of the cannula was verified post-mortem by investigating the spread of ink dye after i.c.v. injection.

Test stimuli were delivered to the Schaffer-collateral/commissural pathway every 30 s to evoke field EPSPs that were 45–60% maximum amplitude. To induce potentiation the following high frequency stimulation (HFS) protocols were used: w100 Hz, consisting of a single series of 10 trains of 10 stimuli at test pulse intensity with an inter-train interval of 2 s; 100 Hz, consisting of a single series of 10 trains of 20 stimuli at test pulse intensity with an inter-train interval of 2 s; 200 Hz, consisting of a single series of 10 trains of 20 stimuli at test pulse intensity with an inter-train interval of 2 s; s200 Hz HFS, consisting of a single series of 10 trains of 20 stimuli at high intensity (75% maximum) with an inter-train interval of 2 s; 400 Hz, consisting of a single series of 10 trains of 20 stimuli at test pulse intensity with an inter-train interval of 2 s; s400 Hz, consisting of a single series of 10 trains of 20 stimuli at high intensity (75% maximum) with an inter-train interval of 2 s; and 3 sets of 10 trains of 20 high intensity (75% maximum) pulses at 400 Hz with an inter-train interval of 2 s and an inter-set interval of 5 min (3 × s400 Hz). To induce depotentiation with electrical low frequency stimulation (LFS), 900 very high intensity pulses (95% maximum) were applied at 1 Hz.

Hippocampal electroencephalogram (EEG) was monitored between recordings of the evoked EPSPs from the same electrodes as described previously (Qi et al., 2013). The power (mV⋅ms) frequency spectrum of theta EEG in the 6–8 Hz theta band was calculated using the modulus of the amplitude (PowerLab Chart version 7 for Windows, ADInstruments Ltd., Oxford, United Kingdom).

Recovery animal experiments were carried out in a well-lit room. The recording compartment consisted of the base of the home cage, including normal bedding and food/water, but the sides were replaced with a translucent Perspex plastic box (27 × 22 × 30 cm) with an open roof. The rats had access to food and water throughout the whole recording session from the same position as in the home cage. All animals were first habituated to the recording procedure over the post-surgery recovery period.

### Novelty Exploration

The novelty exploration protocol used to trigger depotentiation was similar to that described previously (Qi et al., 2013). Briefly, novelty exposure was begun by placing one small elastic ball very gently near the nose of the animal. Once the attention of the animal was drawn to the ball, then the ball was placed on the floor out of immediate reach of the animal. If the animal did not move to explore the ball, the attention-drawing procedure was repeated until the animal moved actively to explore it. Three minutes after the ball was placed, another small object was introduced near the animal using the same attention-drawing method. In order to encourage the rats to continue undisturbed exploration, after four objects, including the ball, had been explored for 12 min in total, clean dark blue tissue paper was inserted gently, totally covering the four walls of the recording compartment and the whole floor including the animal and the objects, leaving animal partly hidden from direct view for another 18 min. The tissue paper and objects were gently removed from the recording compartment at 30 min. In pilot experiments we confirmed (Qi et al., 2013) that such novelty exploration does not persistently affect baseline transmission in WT rats (*n* = 2).

### Drugs

The monoclonal antibody 3A1 was generated by Dr. Brian O’Nuallain against dityrosine cross-linked Aß1–40 with no detectible binding to APP and an ∼700 fold preference for soluble cross-linked Aß aggregates over Aß monomers in Capture/Sandwich ELISA (Frost et al., 2017), and mouse IgG1 isotype control antibody (Biolegend, United Kingdom) were administered in 5 i.c.v. injections (20 μg in 5 μl per injection) over 3 days with the last injection 2 h prior to HFS. We chose this regimen because we found a similar protocol was effective for other anti-Aß strategies in this model. A 20 μg dose of 3A1 was selected because a 10 μg treatment regimen did not reverse the LTP deficit in pilot experiments (*n* = 2, data not shown). Mibefradil (50 nmol in 5 μl i.c.v., Sigma) and (R)-3-(2-carboxypiperazin-4-yl) propyl-1-phosphonic acid (CPP, 7 mg/kg, i.p., Ascent Scientific) were dissolved in distilled water and administered 30 min and 2 h prior to HFS, respectively. The doses were chosen based on their ability to inhibit LTP with different HFS protocols in WT rats (Doyle et al., 1996; Ryan et al., 2010).

### Data Analysis

Unless otherwise stated, the magnitude of potentiation and the power of 6–8 Hz EEG frequency are measured as a percentage of the baseline recordings made during the initial 30-min period, and expressed as the mean ± standard error of the mean. For statistical analysis, EPSP amplitudes were grouped into 10-min epochs. We used standard one-way ANOVA to compare the level of potentiation between multiple groups and one-way ANOVA with repeated measures to compare multiple times within groups. Two-way ANOVA with repeated measures was used to analyse the EEG. A significant overall ANOVA was followed by *post hoc* Bonferroni-corrected *t*-tests. Paired and unpaired Student’s *t*-tests were used to compare potentiation within one group and between two groups, respectively. A *P* < 0.05 was considered statistically significant.

## Results

### Targeting Aß Oligomers Reverses the LTP Deficit in Freely Behaving TG Rats

**FIGURE 2:**
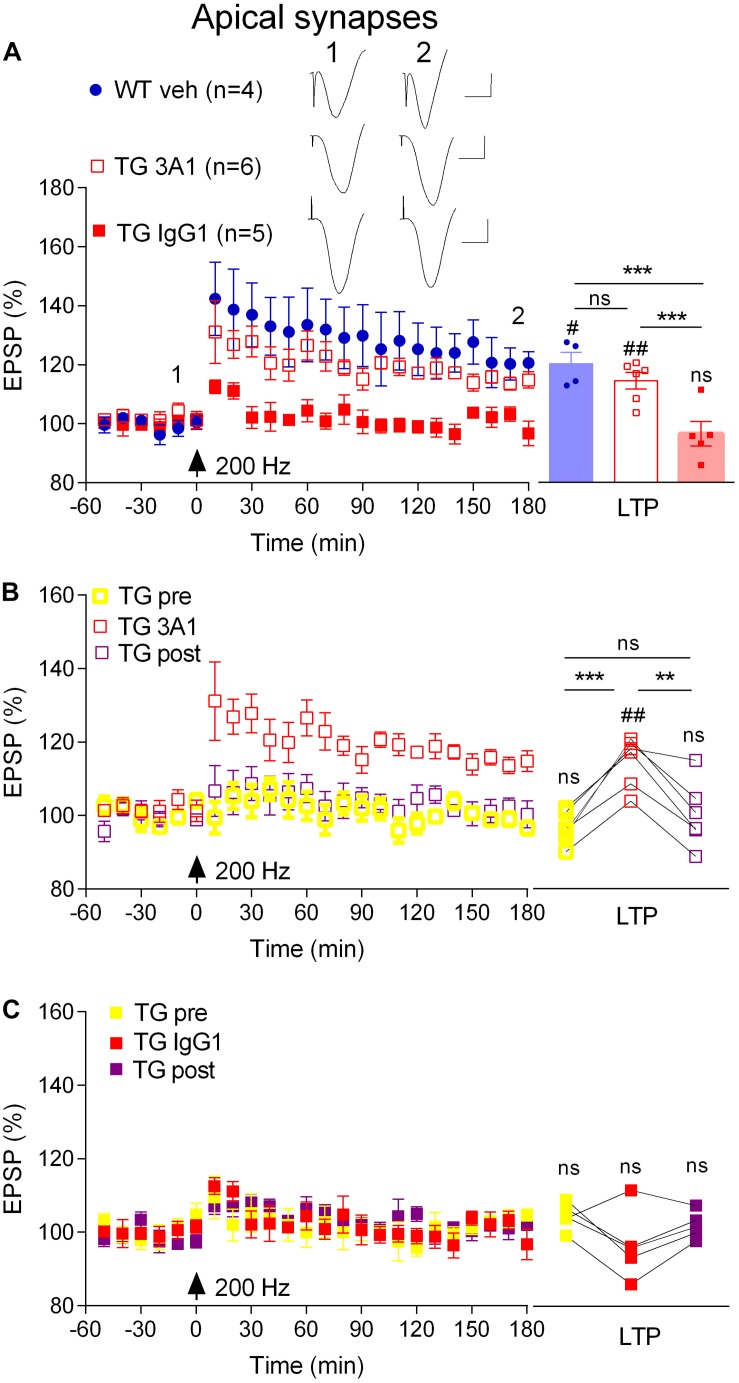
An aggregate conformation-selective anti-Aß antibody, 3A1, transiently rescues the LTP deficit in freely behaving transgenic rats. **(A)** In a cross-sectional analysis, repeated i.c.v. treatment of 4–6-month-old TG rats with 3A1 (TG 3A1) but not isotype control antibody IgG1 (TG IgG1) restored LTP to a level indistinguishable from WT littermates injected with vehicle (WT veh). Left hand panel shows LTP time course. Inserts show representative EPSP traces at the times indicated. Calibration bars: vertical, 1 mV; horizontal, 10 ms. Summary bar chart of LTP (last 10 min post-HFS) is in the right-hand panel. **(B,C)** A longitudinal analysis revealed the transient nature of the recovery of LTP in TG animals injected with 3A1. The LTP time course from the same TG animals the week before (TG pre), immediately after (TG 3A1 or TG IgG1), and 1 week after (TG post) treatment is shown in the left panels. Right hand panels show longitudinal data from individual rats. Arrows indicate the time point of application of a single set of 200 Hz HFS at test pulse intensity (200 Hz). The # symbol stands for a statistical comparison between pre- and 3 h post-HFS values within one group (paired *t*-test) whereas an ^*^ indicates a comparison of 3 h post-HFS values between groups (one-way ANOVA **(A)** or one-way ANOVA with repeated measures **(B,C)** followed by *post hoc* Bonferroni test). One symbol, *p* < 0.05; two symbols, *p* < 0.01; three symbols, *p* < 0.001; ns, *p* > 0.05. Values are mean ± S.E.M.% pre-HFS baseline EPSP amplitude.

Previously we reported that an antibody that recognizes all conformations of Aß, including monomers, soluble aggregates and fibrils, reversed the LTP deficit in TG rats (Qi et al., 2014). Here, we directly examined the involvement of soluble Aß aggregates in mediating the inhibition of LTP at apical synapses between CA3 and CA1 pyramidal cells in 4–6-month-old, pre-plaque, TG rats using the conformation-selective anti-Aß monoclonal antibody 3A1 (Frost et al., 2017). We followed the same 3-day antibody injection protocol that we previously employed to study the Aß-dependence of impaired LTP in these animals (Qi et al., 2014) whereby TG animals received i.c.v. injections of 3A1 or an isotype control antibody IgG1 (5 × 20 μg in 5 μl). Consistent with published findings (Qi et al., 2014), whereas our standard, “200 Hz” induction protocol, consisting of a single set of 200 Hz trains at test pulse intensity, triggered LTP in vehicle-injected freely behaving 4–6-month-old WT rats (3 h post-HFS, 120.6 ± 3.9%, *p* = 0.02 compared with pre-HFS baseline, *n* = 4), the same protocol failed to induce LTP in their TG littermates that had received the control antibody (94.9 ± 2.7%, *p* = 0.42 compared with pre-HFS baseline, *n* = 5, Figure 2A). Unlike the isotype control-treated TG rats, repeated treatment with the conformation-selective anti-Aß antibody 3A1 (5 × 20 μg in 5 μl) reversed the LTP deficit in the TG rats (114.8 ± 2.8%, *n* = 6, *p* = 0.009 compared with baseline, *p* = 0.0007 compared with TG animals injected with IgG1, *p* = 0.25 compared with WT littermates, Figure 2A). When these TG animals were followed longitudinally (Figures 2B,C) it was clear that the recovery of the ability to induce LTP by 3A1 was transient. Thus, LTP was strongly inhibited in these rats when tested again, a week after ceasing treatment with 3A1 (*p* = 0.385, compared with pre-treated animals, *p* = 0.0028, compared with animals immediately after treatment, *n* = 5, Figure 2B). In TG animals treated with the control antibody LTP was inhibited at all time points over the same period (Figure 2C). These findings provide convincing evidence of a requirement for Aßo in mediating LTP inhibition in the TG rats and are consistent with our previous report of an age-dependent accumulation of Aßo in the brains of these animals (Zhang D. et al., 2017).

### Strong High Frequency Stimulation Is Required to Induce Robust LTP in Freely Behaving TG Rats

**FIGURE 3:**
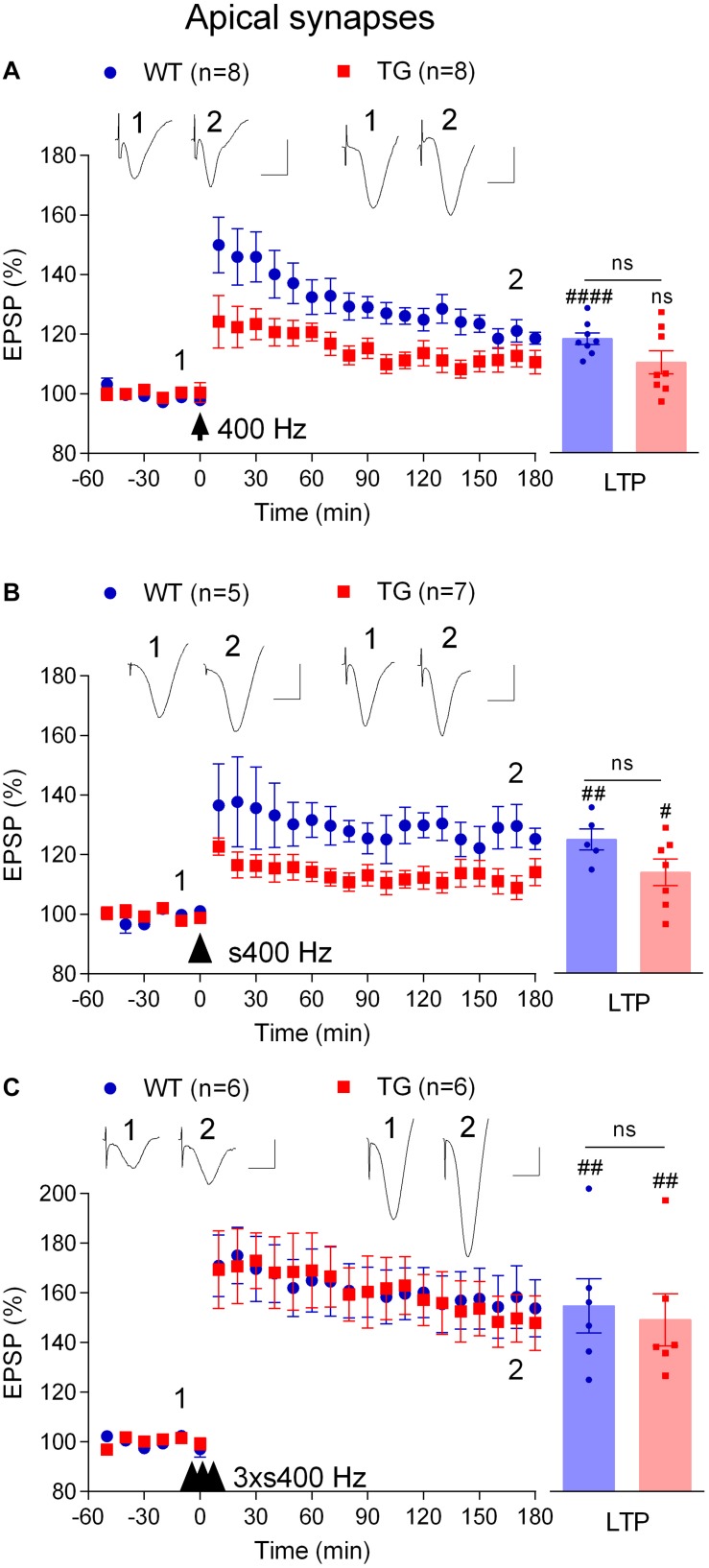
Tetanus strength-dependent potentiation in freely behaving TG rats. To develop a conditioning protocol that would induce robust LTP in TG rats comparable to that found in WT littermates a range of HFS paradigms were applied: **(A)** a single set of 400 Hz trains at 50% of maximal EPSP amplitude (400 Hz); **(B)** a single strong set of 400 Hz trains at 75% of maximal EPSP amplitude (s400 Hz); **(C)** three strong sets of 400 Hz trains at 75% of maximal EPSP amplitude (3 × s400 Hz). Left-hand panels show LTP time course. Inserts show representative EPSP traces at the times indicated. Calibration bars: vertical, 1 mV; horizontal, 10 ms. The time point of HFS application is indicated by arrow, arrow head and three arrow heads, respectively. Summary bar charts of LTP (last 10 min post-HFS) are in right hand panel. The # symbol stands for a statistical comparison between pre- and 3 h post-HFS values within one group (paired *t*-test) whereas an “ns” above the line indicates a comparison of 3 h post-HFS values between groups (unpaired *t*-test). One symbol, *p* < 0.05; two symbols, *p* < 0.01; four symbols, *p* < 0.0001; ns, *p* > 0.05. Values are mean ± S.E.M.% pre-HFS baseline EPSP amplitude.

In order to study depotentiation in TG rats, we needed to generate similar LTP to that induced in WT animals. Previously, we found that increasing the strength of the HFS protocol, using three sets of trains of high intensity pulses at 400 Hz, overcomes the LTP deficit (Qi et al., 2014). We questioned if such a strong protocol was necessary, or if other, intermediate strength, protocols might be sufficient to induce robust LTP in these TG rats. Therefore, we assessed the HFS-dependence of the LTP inhibition in TG rats further by increasing the frequency and intensity. Like 200 Hz HFS, a single train of 400 Hz tetanization at test pulse intensity triggered LTP in 4–6-month-old WT rats (118.6 ± 2.0%, *p* < 0.0001 compared with pre-HFS baseline, *n* = 8) and not significant potentiation in TG littermates (110.6 ± 3.9%, *p* = 0.093, compared with pre-HFS baseline, *n* = 8, Figure 3A). Somewhat similar results were obtained when the same protocol was applied but with high intensity pulses during the tetanus (75% maximum, s400 Hz). However, in this case a significant LTP was induced in both WT (125.3 ± 3.6%, *p* = 0.004 compared with pre-HFS baseline, *n* = 5) and TG rats (114.1 ± 4.5%, *p* = 0.019, compared with pre-HFS baseline, *n* = 7, Figure 3B). In contrast, and consistent with our previous observations (Qi et al., 2014), three sets of 400 Hz at high intensity (3 × s400 Hz) induced large and stable LTP in 4–6-month-old TG rats (147.8 ± 11.0%, *p* = 0.007, compared with pre-HFS baseline, *n* = 6) and their WT littermates (153.8 ± 11.5%, *p* = 0.004 compared with pre-HFS baseline, *n* = 6, Figure 3C).

**FIGURE 4:**
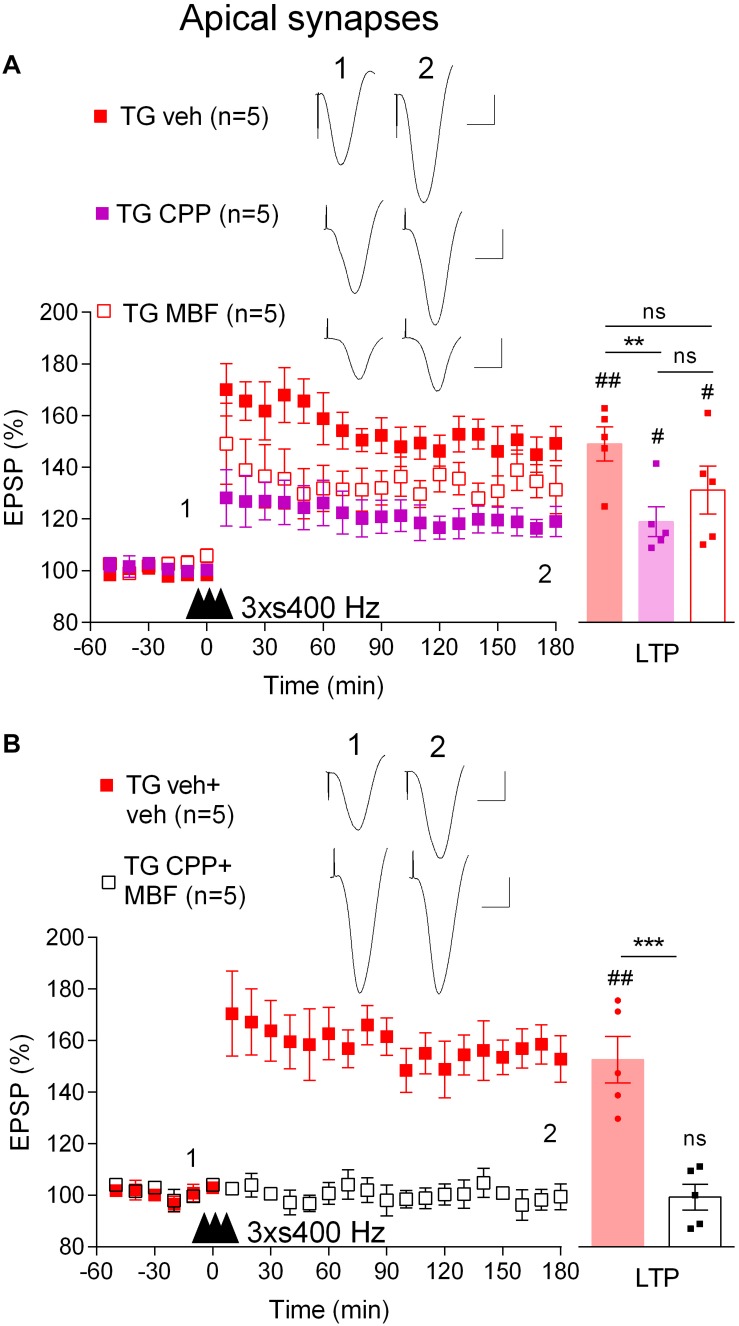
LTP induced by three strong sets of 400 Hz HFS is both NMDA receptor- and VGCC-dependent in freely behaving TG rats. **(A)** Pretreatment with either the NMDA receptor antagonist CPP (2 h prior to 3 × s400 Hz HFS, i.p.) or the VGCC inhibitor mibefradil (MBF, 30 min prior to 3 × s400 Hz HFS, i.c.v.) alone only partly inhibited LTP induction. **(B)** Pretreatment with the combination of these agents completely inhibits LTP in TG animals. Left-hand panels show LTP time course. Inserts show representative EPSP traces at the times indicated. Calibration bars: vertical, 1 mV; horizontal, 10 ms. The time point of HFS application is indicated by three arrow heads. Summary bar charts of LTP (last 10 min post-HFS) are in right hand panel. The # symbol stands for a statistical comparison between pre- and 3 h post-HFS values within one group (paired *t*-test) whereas an ^*^ indicates a comparison of 3 h post-HFS values between groups [one-way ANOVA followed by *post hoc* Bonferroni tests in **(A)** or unpaired *t*-test in **(B)**]. One symbol, *p* < 0.05; two symbols, *p* < 0.01; three symbols, *p* < 0.001; ns, *p* > 0.05. Values are mean ± S.E.M.% pre-HFS baseline EPSP amplitude.

We wondered if the mechanism underlying the induction of LTP by 3 × s400 Hz in TG rats was similar to what we had found previously in WT rats which required activation of voltage-gated voltage-gated Ca^2+^ channels (VGCCs) in addition to NMDA receptors (Ryan et al., 2010). Indeed, pretreatment of TG rats with the NMDA receptors antagonist CPP (7 mg/kg, i.p.) alone only partly reduced the magnitude of LTP (119.0 ± 5.8%, *p* = 0.019, compared with pre-HFS baseline, *n* = 5, *p* = 0.009, compared with vehicle-injected TG, 149.3 ± 6.6%, *n* = 5, Figure 4A). In contrast, LTP was completely blocked when CPP (7 mg/kg, i.p.) and the VGCC inhibitor mibefradil (50 nmol in 5 μl, i.c.v.) were administered in combination (99.4 ± 5.0%, *p* = 0.48, compared with pre-HFS baseline, *n* = 5, Figure 4B). We also confirmed that this combination completely inhibited LTP induced by the 3 × s400 Hz protocol in WT littermates (99.8 ± 4.2%, *p* = 0.43, compared with pre-HFS baseline, *n* = 5, not illustrated).

These data indicate that in order to induce robust LTP in TG rats it was necessary to use a strong HFS protocol that engages VGCCs in addition to the NMDA receptors, in contrast to LTP induced by our standard 200 Hz protocol in WT rats which only requires NMDA receptors (Hu et al., 2008).

### Novelty Exploration-Induced Depotentiation Is Impaired in an Aßo-Dependent Manner in Freely Behaving TG Rats

**FIGURE 5:**
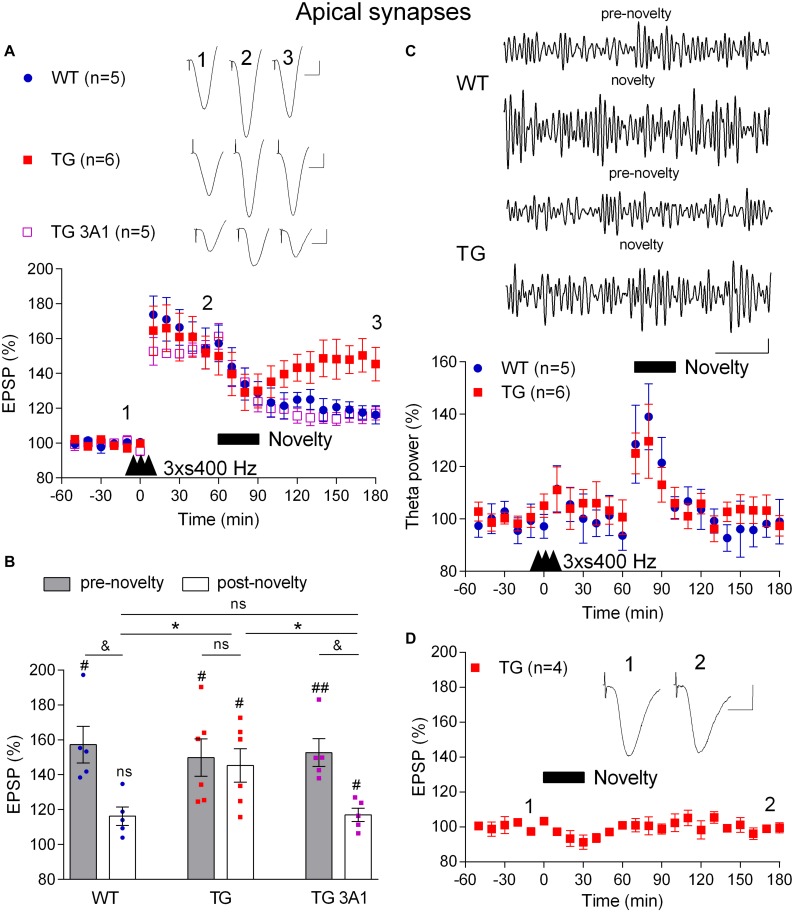
A deficit in novelty exploration-induced depotentiation in freely behaving 4–6-month-old TG rats is reversed by the conformation-selective anti-Aß antibody 3A1. **(A)** 1 h after LTP induction by 3 × s400 Hz the rats were allowed to continuously explore a novel environment for 30 min (Novelty, solid bar). Whereas novelty exploration failed to reverse LTP in TG rats, depotentiation was induced in WT littermates and TG rats previously treated for 3 days with the monoclonal antibody 3A1 (TG 3A1). Inserts show representative EPSP traces at the times indicated. Calibration bars: vertical, 1 mV; horizontal, 10 ms. **(B)** Summary bar chart of the magnitude of potentiation both pre-novelty (50–60 min post-HFS epoch) and post-novelty (170–180 min post-HFS epoch). The # symbol stands for a statistical comparison with pre-HFS values within one group and the & symbol stands for a statistical comparison with pre-novelty values within one group (one-way ANOVA with repeated measures followed by *post hoc* Bonferroni test). An ^*^ indicates a comparison of post-novelty values between groups (one-way ANOVA with *post hoc* Bonferroni test). One symbol, *p* < 0.05; two symbols, *p* < 0.01; ns, *p* > 0.05. The time point of HFS application is indicated by three arrow heads. **(C)** Increased theta band EEG power (6–8 Hz frequency range) during novelty exploration in both WT and TG rats. Top panels show representative examples of EEG band-pass filtered at 5–15 Hz. Calibration bars: vertical, 0.1 mV; horizontal, 1 s. Bottom panel shows time course of EEG changes. **(D)** No discernable change in baseline synaptic transmission after novelty exploration (Novelty, solid bar) in TG rats. Insert shows representative EPSP traces at the times indicated. Calibration bars: vertical, 1 mV; horizontal, 10 ms. Values are mean ± S.E.M.% either pre-HFS baseline EPSP amplitude **(A,B,D)** or theta power **(C)**.

Just like memories, LTP is transiently susceptible to active erasure due to the ability of additional experience or electrical LFS to reverse this form of synaptic potentiation (Kim et al., 2007; Clem and Huganir, 2010; Diaz-Mataix et al., 2011). Given the potential importance of such activity-dependent persistent reversal of previously established synaptic LTP in brain function and our previous findings with exogenous Aß-containing APP fragments (Kim et al., 2001), we wondered if behaviourally or electrically induced depotentiation is also disrupted in TG rats. Prolonged exploration of a non-aversive novel environment can trigger a rapid depotentiation of LTP at CA3-CA1 synapses that is prevented by prior acquisition of information about the new environment (Xu et al., 1998; Manahan-Vaughan and Braunewell, 1999). To compare the ability of novelty exploration to instigate depotentiation in 4–6-month-old WT and TG rats, first we applied the 3 × s400 Hz protocol to induce robust LTP (Figures 5A,B). One h after the induction of LTP the animals were allowed to actively explore novel objects that were placed in the recording box for 30 min. Whereas novelty exploration in the WT rats strongly reversed the previously established LTP (3 h post-HFS, 116.2 ± 5.3%, *p* = 0.006, compared with a pre-novelty potentiation, 157.3 ± 10.5%, *p* > 0.05, compared with pre-HFS baseline, *n* = 5), similar novelty was much less effective in TG rats (3 h post-HFS, 149.9 ± 10.7%, *p* = 0.29, compared with pre-novelty potentiation, 145.3 ± 9.6%, *p* = 0.006, compared with pre-HFS baseline, *n* = 6). In contrast, brief novelty exploration was effective in triggering depotentiation in TG rats that had received repeated i.c.v. injections of the anti-Aßo antibody 3A1 (5 × 20 μg in 5 μl over 3 days) (117.0 ± 3.8%, *n* = 5, *p* = 0.005, compared with pre-novelty potentiation, 161.2 ± 7.3%, *p* = 0.008, compared with pre-HFS baseline, *p* = 0.032 compared with TG animals, *p* = 0.90 compared with WT littermates).

Consistent with a widespread activation of the hippocampus during novelty exploration, theta power of the local EEG was increased, particularly during the first 20 min (Figure 5C) and there was no significant difference in the magnitude of the increase in theta power between WT and TG rats (*p* = 0.33, for the group × time interaction, two-way ANOVA with repeated measures). This indicates that the extent of hippocampal engagement was similar in both groups and therefore unlikely to underlie the difference in the magnitude of depotentiation induced in the two groups. Moreover, similar to our previous finding in WT animals (Qi et al., 2013), novelty exploration did not affect baseline synaptic transmission in TG rats (99.4 ± 3.0%, *p* = 0.304, compared with pre-novelty values, *n* = 4, Figure 5D).

### Age-Dependent Inhibition of Electrically Induced Depotentiation in Anaesthetized TG Rats

In addition to novelty exploration, the application of electrical LFS triggers an NMDA receptor-dependent depotentiation *in vivo* that can be studied under anaesthesia (Doyle et al., 1997). This enabled us to evaluate depotentiation under conditions where possible confounding effects of behavioral phenotype are unlikely to affect the outcome.

**FIGURE 6:**
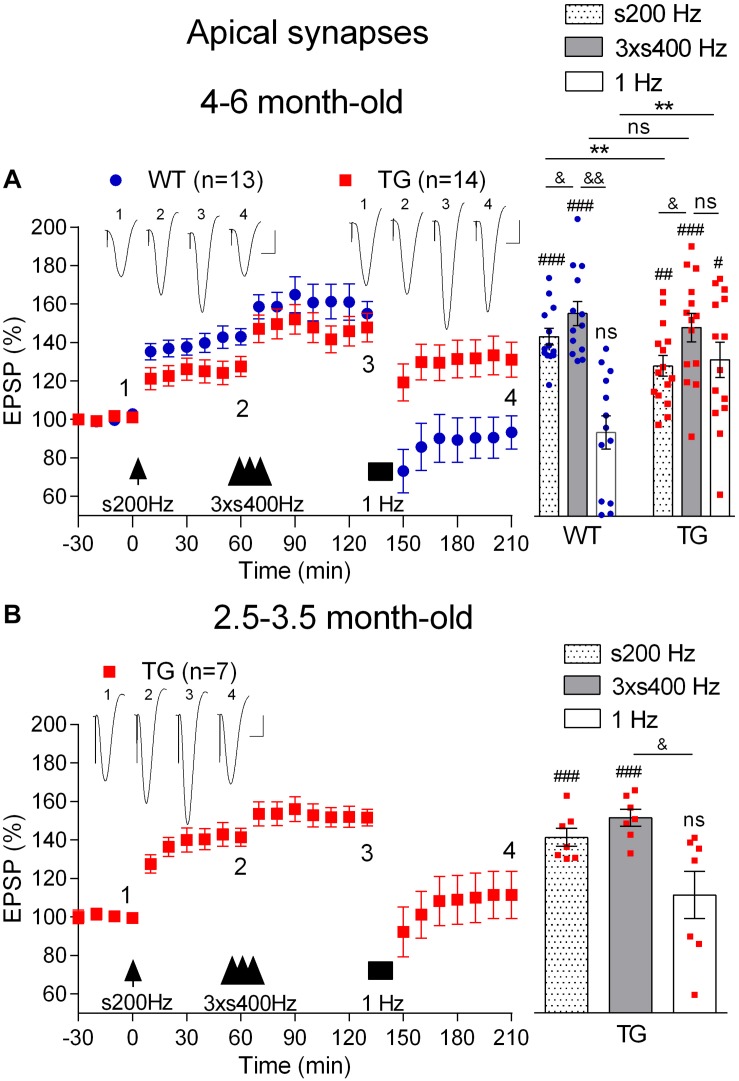
Age-dependent deficit in depotentiation in anaesthetized TG rats. **(A)** Application of 1 Hz LFS (solid bar) following LTP induction by a sequence of s200 Hz HFS (arrow) and 3 × s400 Hz HFS (three arrow heads) failed to induce persistent depotentiation in 4–6-month-old TG rats, unlike their WT littermates, under urethane anaesthesia. In contrast application of the same 1 Hz LFS protocol completely reversed LTP induced by 3 × s400 Hz HFS in younger (2.5–3.5-month-old) TG animals **(B)**. Left hand panels show LTP time course. Inserts show representative EPSP traces at the times indicated. Calibration bars: vertical, 1 mV; horizontal, 10 ms. Summary bar charts of potentiation at the same three time points (s200 Hz, last 10 min post-s200 Hz HFS; 3 × s400 Hz, last 10 min post-3 × s400 Hz HFS; 1 Hz, last 10 min post-1 Hz LFS) are in the right-hand panels. The # symbol stands for a statistical comparison with pre-HFS values within one group and the & symbol stands for a statistical comparison with pre-LFS values within one group (one-way ANOVA with repeated measures followed by *post hoc* Bonferroni test). An ^*^ indicates a comparison of potentiation values between groups (one-way ANOVA with *post hoc* Bonferroni test). One symbol, *p* < 0.05; two symbols, *p* < 0.01; three symbols, *p* < 0.001; ns, *p* > 0.05. Values are mean ± S.E.M.% pre-HFS baseline EPSP amplitude.

First, we confirmed (Qi et al., 2014) that LTP induced by 200 Hz at high pulse intensity (75% maximum, s200 Hz) was inhibited in anaesthetized 4–6-month-old TG rats (1 h post-s200 Hz HFS, 123.6 ± 5.1%, *n* = 14, *p* = 0.009, compared with WT littermates, 143.1 ± 4.3%, *n* = 13, Figure 6A). Following this, we applied the 3 × s400 Hz HFS protocol, which induced LTP that was similar in magnitude in both groups (1 h post-3xs400 Hz HFS, TG, 147.8 ± 7.6%, *p* = 0.47, compared with WT, 155.0 ± 6.3%). One h later we applied LFS consisting of 900 very high intensity pulses (95% maximum) at 1 Hz. As expected, LFS completely and persistently reversed LTP in WT rats (3.5 h post-s200 Hz HFS, 90.3 ± 10.1%, *p* = 0.23, compared with pre-s200 Hz HFS baseline). In contrast, the same 1 Hz protocol in the TG littermates only caused a transient reversal of LTP (3.5 h post-s200 Hz HFS, 131.1 ± 9.1%, *p* = 0.008, compared with pre-s200 Hz HFS baseline).

To determine the possible age-dependence of the deficit in depotentiation in the TG rats, we examined the efficacy of LFS to reverse LTP triggered by either the 3 × s400 Hz HFS or the s200 Hz protocol in 2.5–3.5-month-old TG rats, an age when there is no apparent LTP deficit (Qi et al., 2014). Thus, one h after the induction of LTP with the 3 × s400 Hz HFS protocol in the younger rats the application of LFS triggered a strong reversal of LTP (2.5 h post-3 × s400 Hz HFS, 111.5 ± 12.3%, *p* = 0.41 compared with pre-HFS baseline, *n* = 7, Figure 6B). Similar findings were observed for depotentiation after the s200 Hz protocol (2.5 h post-s200 Hz HFS alone, 96.4 ± 8.1%, *p* = 0.79 compared with pre-HFS baseline, *n* = 6, data not illustrated), indicating that the deficit in depotentiation is age-dependent, with a time of onset similar to the impairment in LTP induction by the 200 Hz HFS protocol at these synapses (Qi et al., 2014).

### LTP and Depotentiation at Basal Synapses Are Resistant to Disruption in Freely Behaving TG Rats

Most research has focused on the disruptive effects of Aß on plasticity at apical synapses between CA3 and CA1 hippocampal pyramidal cells. At a circuit level, different CA1 pyramidal cells have different inputs and outputs and perform multiple tasks in parallel (Slomianka et al., 2011; Soltesz and Losonczy, 2018). This diversity is reflected in the expression of different receptors and types of plasticity at different input synapses (Roth and Leung, 1995; Colavita et al., 2016; Brzdak et al., 2019). Moreover, the susceptibility of LTP at the different synaptic inputs to disruption by Aß varies. Thus, we (Hu et al., 2009) and others (Zhao et al., 2018) found that NMDA receptor-dependent LTP at basal synapses, unlike apical synapses, is resistant to inhibition by exogenously applied Aß.

**FIGURE 7:**
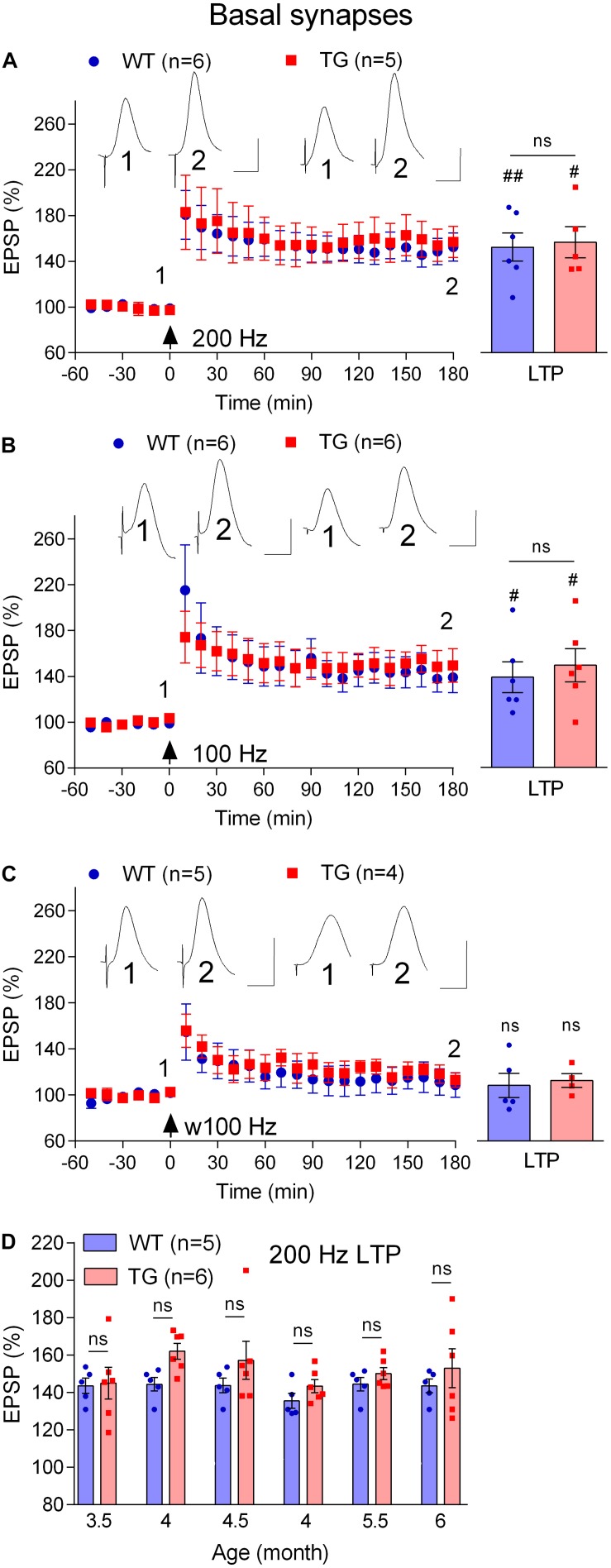
Lack of LTP deficit at basal dendrites in freely behaving TG animals. **(A–C)** Cross-sectional studies of LTP inducibility by different HFS protocols (200 Hz, a standard single set of 200 Hz HFS at test pulse intensity consisting 20 pulses; 100 Hz, a standard single set of 100 Hz HFS at test pulse intensity consisting 20 pulses; w100 Hz, a weak single set of 100 Hz HFS at test pulse intensity consisting 10 pulses) in 4–6-month-old rats. Left hand panels show LTP time course. Inserts show representative EPSP traces at the times indicated. Calibration bars: vertical, 1 mV; horizontal, 10 ms. The time point of HFS application is indicated by an arrow. Summary bar charts of LTP (last 10 min post-HFS) are in the right-hand panel. **(D)** Summary longitudinal data for the magnitude of 200 Hz HFS induced LTP tracked repeatedly in the same WT and TG rats every 2nd week between 3.5 and 6 months of age. The # symbol stands for a statistical comparison between pre- and 3 h post- HFS values within one group (paired *t*-test) whereas an “ns” above the line in **(A–C)** and throughout **(D)** indicates a comparison of 3 h post-HFS values between groups (unpaired *t*-test). One symbol, *p* < 0.05; two symbols, *p* < 0.01; ns, *p* > 0.05. Values are mean ± S.E.M.% pre-HFS baseline EPSP amplitude.

First, we compared the ability of the standard 200 Hz conditioning stimulation protocol to trigger LTP at basal synapses in 4–6-month-old WT and TG freely behaving rats, an age when LTP is inhibited at apical synapses. With the stimulation electrode in stratum oriens, the far-field EPSP from basal synapses was reversed in polarity because the recording electrode was located in the stratum radiatum (Leung et al., 2003; see Figure 1). Different from apical synapses, the application of HFS at basal synapses induced similar magnitude of LTP in both groups (WT, 152.6 ± 12.4, *p* = 0.004, compared with pre-HFS baseline, *n* = 6, TG, 156.9 ± 13.6, *p* = 0.017, compared with pre-HFS baseline, *n* = 5, WT versus TG, *p* = 0.82, Figure 7A). Similarly, there was no evidence of inhibition of LTP when we tested rats longitudinally between 3.5 and 6 months (*p* > 0.05 for all ages tested, Figure 7D). Because we were primarily interested in the effects of pre-fibrillar soluble Aß aggregates, we did not investigate animals older than 6 months, when Aß plaques start to be detectible in some TG rats (Leon et al., 2010; Hanzel et al., 2014; Iulita et al., 2014).

Amongst other differences, the threshold for LTP induction at basal synapses has been reported to be lower at basal compared with apical synapses (Leung et al., 2003). Because the LTP deficit at apical synapses was associated with an apparent increase in threshold, we decided to assess if the threshold for LTP was altered at basal synapses using weaker HFS protocols. When the frequency of the conditioning stimulation was reduced from 200 Hz to 100 Hz, similar magnitude LTP was induced both in 4–6-month-old TG and WT littermates (WT, 139.0 ± 13.5, *p* = 0.04, compared with pre-HFS baseline, *n* = 6, TG, 149.5 ± 14.7, *p* = 0.028, compared with pre-HFS baseline, *n* = 6, Figure 7B). Moreover, in order to determine if LTP might be facilitated in stratum oriens in the TG rats, we reduced the number of pulses per train from 20 to 10 (w100 Hz). This very weak HFS didn’t induce LTP in either group (WT, 108.7 ± 10.6, *p* = 0.59, compared with pre-HFS baseline, *n* = 5, TG, 113.0 ± 6.2, *p* = 0.24, compared with pre-HFS baseline, *n* = 4, Figure 7C).

**FIGURE 8:**
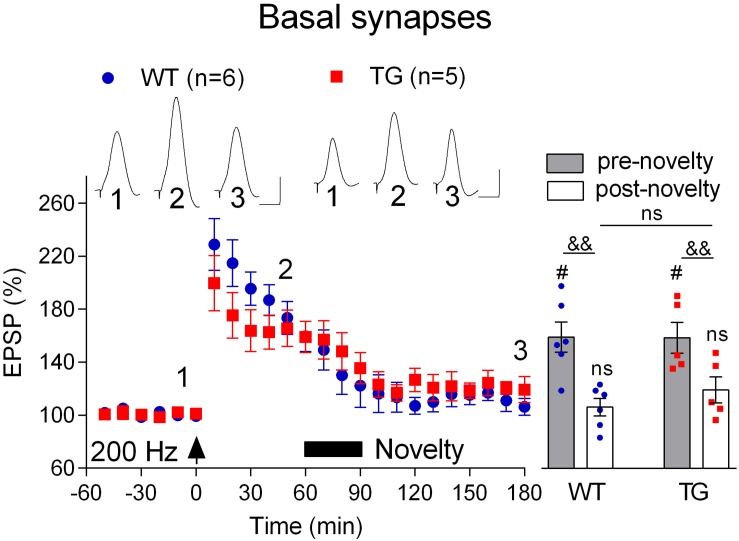
Depotentiation is normal at basal dendrites in freely behaving TG animals. One hour after LTP induction by 200 Hz conditioning stimulation 4–6-month-old rats were allowed to continuously explore a novel environment for 30 min (Novelty, solid bar). Left hand panels show the time course of LTP and depotentiation. Inserts show representative EPSP traces at the times indicated. Calibration bars: vertical, 1 mV; horizontal, 10 ms. Summary bar chart of pre-novelty (pre, 50–60 min post-HFS epoch) and post-novelty (post, 170–180 min post-HFS epoch) potentiation is in right hand panel. The # symbol stands for a statistical comparison with pre-HFS values within one group and the & symbol stands for a statistical comparison with pre-novelty values within one group (one-way ANOVA with repeated measures followed by *post hoc* Bonferroni test). An “ns” above the line indicates a comparison of post-novelty values between groups (one-way ANOVA with *post hoc* Bonferroni test). One symbol, *p* < 0.05; two symbols, *p* < 0.01; ns, *p* > 0.05. The time point of HFS application is indicated by an arrow. Values are mean ± S.E.M.% either pre-HFS baseline EPSP amplitude.

Finally, we wondered if, like LTP, novelty exploration-induced depotentiation at the basal synapses (Qi et al., 2013) is preserved in freely behaving 4–6-month-old TG rats. Consistent with previous studies (Hu et al., 2009; Qi et al., 2013), the initial post-HFS potentiation appears decremental. Nevertheless, as seen in Figure 7A, stable LTP was recorded in both WT and TG rats during the subsequent 2 h post-200 Hz HFS. In contrast, LTP reverted back to baseline when rats were allowed explore novel objects for 30 min, starting 1 h after inducing LTP with the 200 Hz protocol. Thus, the magnitude of depotentiation was similar in both groups (WT, 106.4 ± 6.5, *p* = 0.30, compared with pre-HFS baseline, *n* = 6, TG, 119.4 ± 9.8, *p* = 0.16, compared with pre-HFS baseline, *n* = 5, Figure 8).

## Discussion

Here we provide evidence that, prior to plaque deposition, Aßo mediate an age-dependent inhibition of both LTP and depotentiation at apical synapses in the CA1 area of APP TG rats. To allow us to study depotentiation in these rats we used a strong protocol to induce an additional LTP that was blocked by combined treatment with an NMDA receptor antagonist and a VGCC inhibitor. Novelty exploration in freely behaving animals and electrical LFS under anaesthesia failed to trigger depotentiation at apical synapses at the pre-plaque stage. In contrast, neither LTP nor novelty exploration-induced depotentiation was altered at basal synapses in similarly aged TG rats. Thus, the age-dependent deficit in LTP and depotentiation is selective for apical synapses. This differential vulnerability of plasticity at apical and basal synapses strongly indicates a circuit-selective reduction in the dynamic range of synaptic gain and weakening.

The ability of repeated dosing with an Aßo-selective antibody, 3A1 (Frost et al., 2017) provides evidence that the age-dependent LTP deficit in pre-plaque TG rats is mediated by Aßo. This finding extends our previous reports that (a) soluble Aß is necessary for the LTP inhibition (Qi et al., 2014), and (b) there is an age-dependent increase in Aßo in the brain of TG rats starting around the time of the onset of the LTP deficit (Zhang D. et al., 2017). Similar to the beneficial action of the non-conformation-selective anti-Aß antibody McSA1 (Qi et al., 2014), when followed longitudinally in individual rats, the LTP impairment re-emerged within 1 week of ceasing treatment with 3A1.

The inhibition of LTP induction by our standard 200 Hz conditioning protocol, which is NMDA receptor-dependent (Doyle et al., 1996; Hu et al., 2008), was hypothesized to be due to an increase in the threshold for LTP induction consequent to a reduction in NMDA receptor-mediated synaptic transmission in TG rats (Qi et al., 2014). Consistent with this proposal, in the present studies whereas intermediate strength protocols were ineffective, repeated high intensity 400 Hz HFS triggered robust LTP in the TG rats. This finding contrasts with our previous report that acute exogenously applied Aßo potently inhibited LTP induced by comparable 200 and 400 Hz conditioning protocols (Klyubin et al., 2014). Similar to WT rats (Doyle et al., 1996; Hu et al., 2008; Ryan et al., 2010), a combination of an NMDA receptor antagonist and VGCC blocker fully prevented LTP induction by repeated high intensity 400 Hz tetanus in TG rats. Since this protocol, unlike the weaker protocols, triggered similar magnitude LTP in both sets of animals, it is possible that VGCC-dependent LTP is relatively spared compared to NMDA receptor-dependent LTP in TG rats. Thus, the inhibition of LTP by endogenous Aßo in TG rats may depend on the source of the initial Ca^2+^ entry trigger for plasticity induction. Future *in vitro* studies with saturating concentrations of selective antagonists will be required to evaluate this possibility.

Contrary to our predictions based on the synaptic weakening-promoting acute effects of exogenously applied Aß (Li et al., 2009; Hu et al., 2014; O’ Riordan et al., 2018) and Aß-containing APP fragments (Kim et al., 2001), depotentiation was strongly inhibited at apical synapses in TG rats. This was the case for both novelty exploration and LFS-induced depotentiation in freely behaving and anaesthetized 4–6-month-old TG rats, respectively. It appears that, in addition to LTP inhibition, Aßo mediate the inhibition of depotentiation in the TG rats since the monoclonal antibody 3A1 also reversed this deficit. The age-dependent increase in Aßo (Zhang D. et al., 2017) and similar age-dependence of the depotentiation and LTP deficits supports a similar role of Aßo in both deficits. In view of the known NMDA receptor-dependence of both novelty exploration- and electrical LFS-induced depotentiation (Doyle et al., 1996; Qi et al., 2013), it seems likely that the deficit in depotentiation in TG rats is, like that suggested for the LTP deficit, mediated by a reduction in NMDA receptor-mediated transmission (Qi et al., 2014).

In order to study depotentiation in TG animals, we needed to first apply the strong, repeated train of 400 Hz protocol to induce robust LTP. Although LTP has been reported to be resistant to activity-dependent reversal when induced by repeated stimulus trains *in vitro* (Woo and Nguyen, 2003), we found that high intensity LFS *in vivo* induced persistent reversal of LTP triggered either by our standard 200 Hz or the strong 400 Hz protocols in younger TG rats. Further studies are required to determine if depotentiation depends on the LTP induction and/or LTP reversal protocols *in vivo*. Based on available evidence, LTP may be susceptible to depotentiation over a longer time period *in vivo* (Doyle et al., 1997; Xu et al., 1998). Given our finding that exploration-induced depotentiation was associated with enhancement of theta power, it would be particularly interesting in future studies to examine if LFS with different frequencies in the theta range are differentially affected in TG rats.

In apparent contrast to the present findings, depotentiation has been reported to be normal in hippocampal slices from young pre-plaque APP TG (Tg2576) mice (Huh et al., 2016). In that study depotentiation induced either by a 5 Hz electrical stimulation protocol (applied 5 min after HFS) or the receptor kinase ErbB4 ligand neuregulin 1, was impaired only in plaque-laden mice. The deficit in the older mice was attributed to damage to certain interneurons that express ErbB4. Whether or not similar changes are present in pre-plaque TG rats is not known. Moreover, because Aß may bind ErbB4 and its ablation prevents exogenously applied Aß-mediated inhibition of LTP (Zhang H. et al., 2017), future studies should further examine its role in depotentiation deficits at the pre-plaque stage of TG rats.

A corollary of the widely accepted theory that LTP-like persistent synaptic strengthening provides an essential component of memory formation is that depotentiation will promote memory erasure. Indeed, interventions, including novelty exploration, that relatively selectively induce depotentiation can trigger the erasure of newly formed memories or habits (Hayashi-Takagi et al., 2015; Medina, 2018; Ge et al., 2019). Investigating whether patients with early AD have a deficit in memory interference from novel information is a topic worth pursuing (Muecke et al., 2018; Thomas et al., 2018).

Our finding that LTP at basal, as opposed to apical, synapses appear to be unchanged in the pre-plaque TG rats is consistent with previous reports that exogenously applied Aßo fails to inhibit LTP in stratum oriens (Hu et al., 2009; Zhao et al., 2018). Recently, it has become clear that CA1 pyramidal neurons with cell bodies either near the stratum radiatum or stratum oriens generally form different networks, with the latter having much more extensive basal dendritic trees with a strong input from CA2 (Graves et al., 2012; Soltesz and Losonczy, 2018). The known different signaling pathways mediating LTP at these synapses (Roth and Leung, 1995; Colavita et al., 2016; Brzdak et al., 2019) and the finding that spines have high turnover rates in stratum oriens (Pfeiffer et al., 2018), may help explain the relative resistance of synaptic plasticity at basal synapses to disruption of both LTP and depotentiation. A significant but relatively poorly explored question for AD research is to understand why only certain pathways are affected early in the disease process (Fu et al., 2018). Understanding the mechanisms underlying the pathway selectivity of the plasticity disrupting action of endogenously generated Aßo, as reported here, may help clarify the early pathophysiology of Alzheimer’s disease.

## Data Availability

The datasets generated for this study are available on request to the corresponding author.

## Ethics Statement

The animal study was reviewed and approved by Animal Research Ethics Committee, Trinity College Dublin and Health Products Regulatory Authority, Ireland.

## Author Contributions

YQ and IK performed the experiments. IK and MR wrote the manuscript. All the authors contributed to study design, and read and approved the final manuscript.

## Conflict of Interest Statement

The authors declare that the research was conducted in the absence of any commercial or financial relationships that could be construed as a potential conflict of interest.
